# The Relationship between Health Literacy and Health Disparities: *A Systematic Review*

**DOI:** 10.1371/journal.pone.0145455

**Published:** 2015-12-23

**Authors:** Sarah Mantwill, Silvia Monestel-Umaña, Peter J. Schulz

**Affiliations:** Institute of Communication & Health, University of Lugano, Lugano, Switzerland; Catholic University of Sacro Cuore, ITALY

## Abstract

**Objectives:**

Health literacy is commonly associated with many of the antecedents of health disparities. Yet the precise nature of the relationship between health literacy and disparities remains unclear. A systematic review was conducted to better understand in how far the relationship between health literacy and health disparities has been systematically studied and which potential relationships and pathways have been identified.

**Methods:**

Five databases, including PubMed/MEDLINE and CINAHL, were searched for peer-reviewed studies. Publications were included in the review when they (1) included a valid measure of health literacy, (2) explicitly conceived a health disparity as related to a social disparity, such as race/ethnicity or education and (3) when results were presented by comparing two or more groups afflicted by a social disparity investigating the effect of health literacy on health outcomes. Two reviewers evaluated each study for inclusion and abstracted relevant information. Findings were ordered according to the disparities identified and the role of health literacy in explaining them.

**Results:**

36 studies were included in the final synthesis. Most of the studies investigated racial/ethnic disparities, followed by some few studies that systematically investigated educational disparities. Some evidence was found on the mediating function of health literacy on self-rated health status across racial/ethnic and educational disparities, as well as on the potential effect of health literacy and numeracy on reducing racial/ethnic disparities in medication adherence and understanding of medication intake.

**Conclusion:**

Overall the evidence on the relationship between health literacy and disparities is still mixed and fairly limited. Studies largely varied with regard to health(-related) outcomes under investigation and the health literacy assessments used. Further, many studies lacked a specific description of the nature of the disparity that was explored and a clear account of possible pathways tested.

## Introduction

Health disparities are differences in health that occur due to social, economic or environmental disadvantages. In particular groups that are more likely to fall victim of discrimination or segregation often face increased difficulties in preserving their health [1]. Disparities in health are mostly measured by comparing two or more groups to each other or to a reference group in general. Likely a disadvantaged group is compared to a more advantaged group, using an indicator of health or health-related outcome [2, 3]. In the United States (US) for example, studies have shown that those with lower education, less income and individuals from ethnic/racial minorities are more often afflicted by worse health compared to more socially advantaged groups [4, 5].

In the field of health literacy, often defined as the “degree to which individuals have the capacity to obtain, process and understand basic health information and services needed to make appropriate health decisions” [6], one does frequently find the underlying assumption that health literacy might explain some of the variation in health disparities that would be otherwise linked to other socioeconomic factors, such as education or income for example [7, 8]. Indeed health literacy has been found to be associated with many of the drivers of health disparities. Studies in the US have shown that non-Whites have more often limited health literacy than Whites [9–11]. Also factors such as lower income or education have been found to be associated with lower levels of health literacy [12, 13]. Thus suggesting that individuals likely to fall victim to social disparities, which in turn lead to worse health outcomes, are also more likely to have lower levels of health literacy.

Healthcare practitioners and researchers, as well as policy makers, have recognized the need to focus on health literacy as a potential intervenable factor by which health disparities can potentially be reduced [14, 15]. However, the precise nature of the relationship between health literacy and health disparities remains unclear. Consequently, also potential explanatory pathways and conceptualizations on how health literacy contributes to disparities remain rather vague.

Adding to Berkman and colleagues’ work [9] the aim of this systematic review was to evaluate to which extent research so far has systematically investigated the relationship between health literacy and health disparities, and whether potential relationships and pathways have been identified. In doing so, the review not only sought to contribute to a better theoretical understanding on how health literacy contributes to disparities, but also to identify gaps and missing links that might warrant further investigation, and to better understand potential leverage points for research, as well as for interventions that aim at reducing disparities.

## Methods

### Search strategy and inclusion criteria

A review protocol was developed and reviewed by two experts in the field of health literacy and health disparities. This review followed the Preferred Reporting Items for Systematic Reviews and Meta-Analyses (PRISMA) [16] (S1 Appendix). To identify relevant published articles the following databases were systematically searched: Cochrane Library, Cumulative Index to Nursing and Allied Health Literature (CINAHL), Educational Resources Information Center (ERIC), PsycInfo and PubMed/Medline. Searches were not limited to any specific time frame or specific language.

The search terms for “health disparities” included related concepts such as “inequality”, “race”, “minority” or “gender”, which had been identified previously using a scoping search. For “health literacy” also the term literacy in combination with “health” was separately included. In addition, the search term “numeracy” was included. When navigating the healthcare system people do not only need to have the ability to read and understand written medical information but also often have to interpret information that is presented in numerical form, such as information in a table or dosage instructions [17].

**Fig 1:**
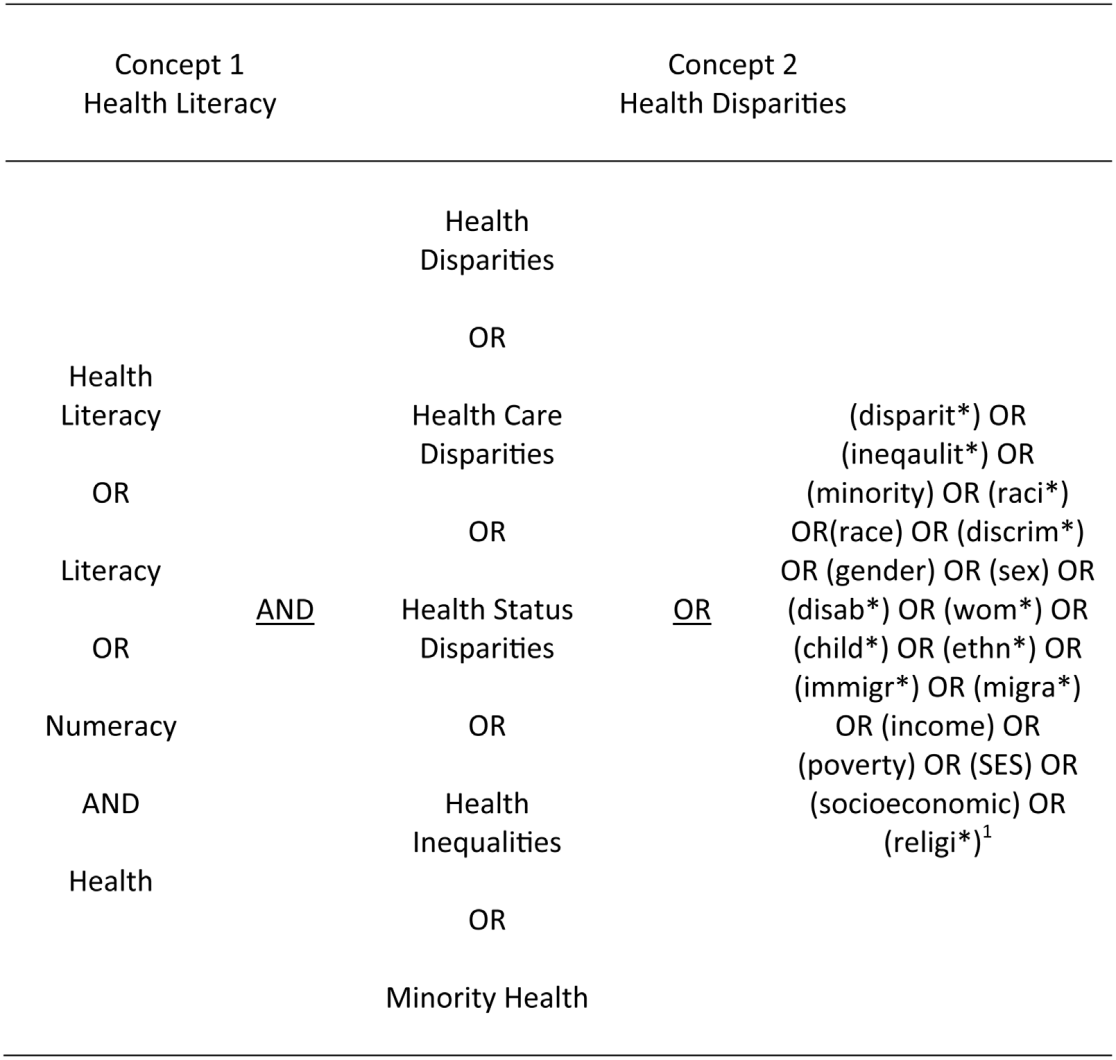
Overview of search strategy. ^1^For demonstration purposes only the last search terms include Boolean operators.

Truncations (wildcard searches) (*), hyphens and other relevant Boolean operators were used to make the search as sensitive as possible (Fig 1). Appropriate MeSH terms were used for searches in PubMed (S2 Appendix). Electronic searches were supplemented by hand searches, and search alerts were set until February 2015. Reference lists of the included articles were further reviewed to identify remaining studies.

Articles were included when they (1) were peer-reviewed (dissertations excluded), (2) included a valid measure of health literacy (direct or indirect), (3) explicitly conceived a health disparity as related to a social disparity/disadvantage, such as race, ethnicity, education or gender and (4) when results were presented by comparing two or more groups afflicted by a social disparity investigating the effect of health literacy on health outcomes.

Measures of health literacy were considered valid if they had been previously tested and had shown construct and/or criterion validity. In addition, measures that showed to have sufficient face validity and were validated in the study under investigation were also considered. It was *not* sufficient if, for instance, a difference in health literacy levels between two racial groups was reported as a secondary outcome. Furthermore, age was not considered to be a predictor of health disparities because certain differences are natural and are likely to occur for other reasons than being socially, economically, or environmentally disadvantaged [2, 18]. Examples are differences between different work positions of which one is more prone to accidents than the other or, in this case, differences between younger and older populations [18].

Any observational study, including cross-sectional, cohort and case-control, examining the relationship between health literacy and health disparities was considered, as well as any experimental study testing for disparities with regard to health literacy. Studies had to report on the association between the disparity under investigation and health literacy. Studies that measured solely disease knowledge were excluded.

### Screening process

After having extracted relevant abstracts from the databases, one reviewer screened all abstracts and titles for duplicates. In a second step two reviewers screened the abstracts for relevance to be included in the review. Discrepant assessments were resolved by discussion. Full manuscripts were retrieved for those abstracts that were identified to be relevant. Two reviewers independently extracted data from the selected studies, using a pre-designed data extraction form, which had been piloted before. Data were synthesized for final analysis and systematically screened by the two reviewers to ensure the correctness of the information. Based on criteria defined by Berkman and colleagues [9], the same reviewers independently rated the quality of articles. Studies were evaluated either as good, fair or poor. The quality assessment tool took such things as selection bias, measurement bias and confounding variables into consideration. Also here disagreement was resolved by consensus finding.

Given that study characteristics varied with regard to health literacy measures, health outcomes, sample sizes and characteristics, it was not deemed feasible to carry out a meta-analysis. Therefore a narrative synthesis was undertaken.

As initially described, disparities in health are mostly measured by comparing two or more groups to each other using an indicator of health(-related) outcome. Therefore findings were ordered according to the health(-related) outcomes that were under investigation in the different included studies. This includes, for instance, self-reported health, cancer-related outcomes or outcomes related to disease control. The social disparity, such as race/ethnicity or gender, as related to the potential health disparity was further reported and the role of health literacy in explaining the disparity under investigation described. Only the association between health literacy and the relevant outcome variables was extracted, even though some of the studies might have reported on additional relationships.

## Results

**Fig 2:**
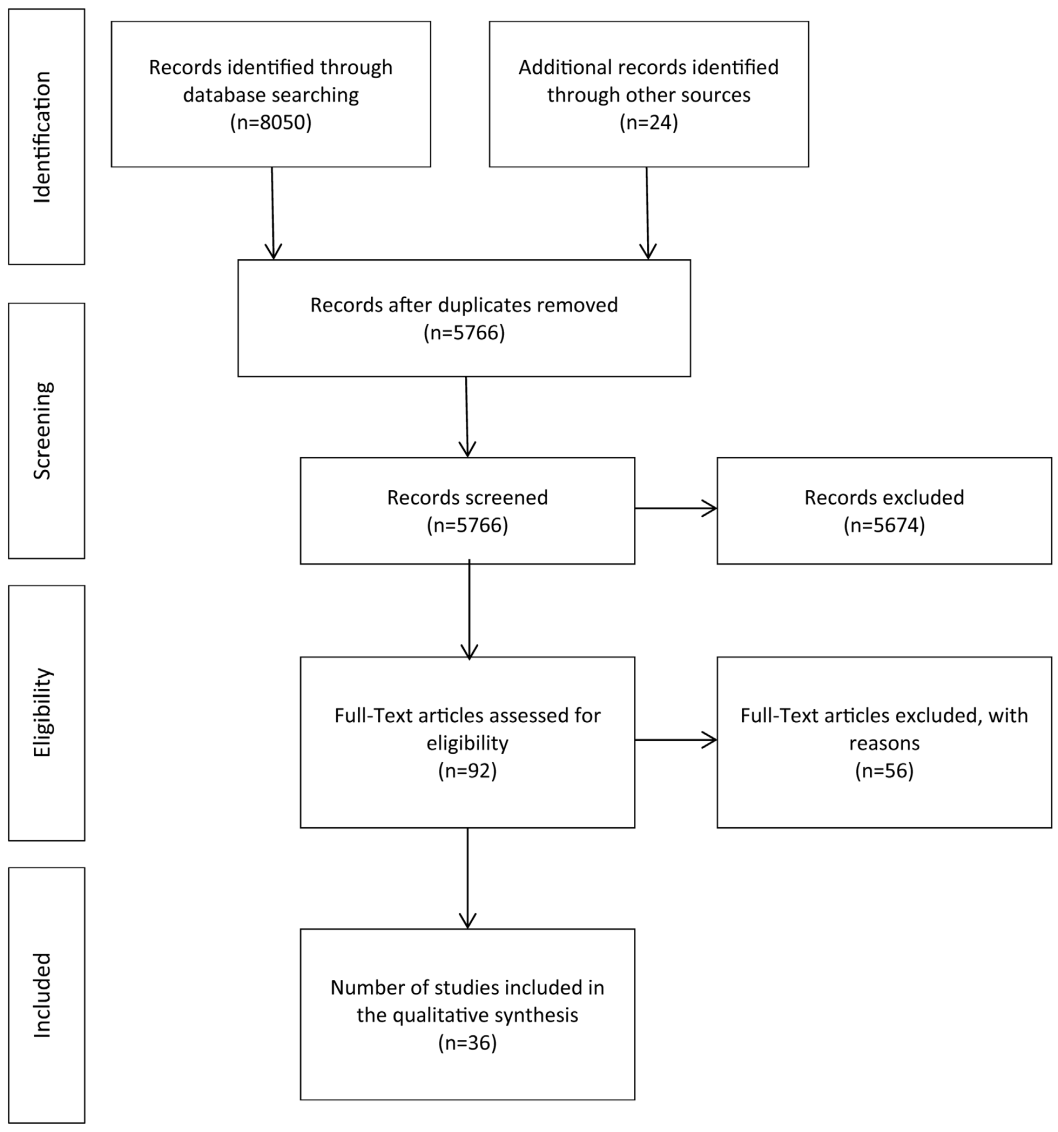
Flowchart of screening process (adapted from: Moher, D., Liberati, A., Tetzlaff, J., & Altman, 2009).

After the removal of duplicates, 5766 abstracts were reviewed and 92 articles were included for full revision. 36 articles were included in the final synthesis (Fig 2). The authors are aware that other studies [19–24] have looked at similar relationships. However, it was decided to exclude them, as they did not sufficiently elaborate or did not explicitly acknowledge the relationship under investigation.

Most studies had been conducted in the US, except for five studies that had been conducted in Canada, China, the Netherlands and respectively the UK.

The following health(-related) outcomes were identified: (1.) self-reported health status, (2.) cancer-related outcomes, (3.) medication adherence/management, (4.) disease control, (5.) preventive care, and (6.) end-of-life decisions. Other individual outcomes, such as usage of complementary and alternative medicine or BMI, were also identified and were grouped into (7.) “other health outcomes”.

Most of the studies focused on race/ethnicity as a social disparity leading to disparities in health(-related) outcomes. For example, eight out of the 36 studies focused on racial/ethnic and educational differences and investigated the influence of health literacy on disparities in self-reported health. Six studies looked into how health literacy might explain cancer-related outcomes that are potentially related to racial/ethnic disparities and another six studies investigated the impact of health literacy and numeracy on medication adherence and management with regard to race/ethnicity.

Some of the studies that were included in this review used multiple health literacy measures. However, the most commonly used measure was the Rapid Estimate of Adult Literacy (-Revised) (REALM-R) [25], which was applied in 15 studies. This was followed by the (Short)-Test of Functional Health Literacy (S-TOFHLA) [26], which had been used in 10 studies. Other measures included the Health Activities and Literacy Scale (HALS) [27] and the health literacy items in the National Assessment of Adult Literacy (NAAL) [10]. Only two studies used the screeners for limited health literacy developed by Chew and colleagues [28], as well as the Newest Vitale Sign (NVS) [29]. Only five studies were identified that tested numeracy skills.

Overall the quality was rated for 16 studies as “good”, 19 were rated as “fair” and only one study was considered to be of “poor” quality (Table 1).

**Table 1:** Overview of included studies.

**Self-Reported Health Status**								
**First Author**	**Design**	**Participants**	**Country**	**Instrument; Cut-off points**	**Sample**	**Outcomes assessed**	**Associations**	**Quality**
**Race/Ethnicity**								
Lee, 2015	Cross-sectional	**White**: 91%, **Asian American Immigrants**: 9% (Chinese (3%), Koreans (2%), Filipinos (2%), South Asians (1%), Vietnamese (1%)	USA	English, Spanish, Chinese (Mandarin, Cantonese), Korean, and Vietnamese: **two subjective proxy measures of health literacy:** continuous.	2007 California Health Interview Survey (CHIS) (N = 33,668)	**Health status**; **Symptoms of depression**	HL was associated with health status and depression symptoms among Whites and aggregated Asian immigrants groups (*p* < .01). In Chinese and Koreans HL was a predictor of self-rated health status (*p* < .05). In Koreans and South Asians HL was a sig. predictor of depression symptoms (*p* < .05).	F
Omariba, 2011	Cross-sectional	**Non-immigrants**: 83% (second generation Canadians: 14.8%), **Immigrants**: 17% (established European or American: 33.1%, established from other countries: 42.6%, recent European or American: 4.6%, recent from other countries: 19.7%)	Canada	English & French **Health Activities Literacy Scale (HALS)** (191 items): low vs. high	Participants International Adult Literacy and Skills Survey (IALSS), ≥16 years (N = 22,818)	**Health status**	Among immigrants the effect of HL on good self-rated health was reduced to n.sig. by discordance between mother tongue and language of survey administration (OR 0.65; 95% CI, 0.45–0.95).	F
Omariba, 2014	Cross-sectional	**First**: 17%, **Second**: 12%, **Third-plus generation immigrants and non-immigrants**: 71%	Canada	English & French **Health Activities Literacy Scale (HALS)** (191 items): low vs. high	Participants International Adult Literacy and Skills Survey (IALSS), ≥16 years (N = 22,818)	**Disability**	HL was n.sig. associated with self-reported disability among different immigrant groups. Among different generations of immigrants a sig. association was found but education, income and employment reduced its effect to n.sig.	F
Sentell, 2011	Cross-sectional	**White**: 34%, **Japanese**: 24.5%, **Filipino**: 14.2%, **Native Hawaiians**: 15.7%, **other AA/PI** (Asian Americans/Pacific Islanders): 11.6%	USA	English & Spanish **Single Health Literacy Screener** ^1^: low vs. adequate	2008 Hawai`i Health Survey (HHS) (N = 4,399)	**Health status**; **Depression**; **Diabetes**; **BMI**	Low HL was associated with poor health status in Japanese, Filipinos, other AA/PI and Whites; with diabetes in Hawaiians and Japanese; and with depression in Hawaiians (*p* < .05). No sig. relationship between HL and being overweight was found.	F
Sentell, 2012	Cross-sectional	**White**: 49% (<1% LEP), **Vietnamese**: 1.5 (38.5% LEP), **Korean**: 1.2% (39.3% LEP), **Chinese**: 3.5% (27.4% LEP), **Latino**: 21.6% (37.3% LEP), **Other ethnicity**: 23.1% (15.2% LEP)	USA	English, Spanish, Chinese (Mandarin, Cantonese), Korean, and Vietnamese: **two subjective proxy measures of health literacy**: continuous	2007 California Health Interview Survey (CHIS) (N = 48,427)	**Health status**	Low HL was only sig. associated with poor health status in White and “other” participants (*p* < .05). LEP was a more important predictor of poor health status in Latinos, Vietnamese, and Whites. Highest odds of poor health status when low HL and low English proficiency combined in Latino, Chinese, Vietnamese and Others.	F
Wang, 2013	Cross-sectional	**Hui minority**: 57.5%, **Han majority**: 42.5%	China	Chinese HL instruments based on revisions of the **Chinese Adult Health Literacy Questionnaire (CAHLQ)**: low vs. high	Field survey in Northwestern China (N = 913)	**Health-related quality of life**	In the Hui group, low HL was a sig. predictor of prevalence pain/discomfort impairments (PR 1.8830, 95% CI 1.06–1.58) but not for the Han group. For anxiety/depression the interaction effect of HL and with ethnic was sig. (*p* < .05).	F
**Education**								
van der Heide, 2013	Cross-sectional	**preprimary or primary education**: 5.5%, **lower secondary education**: 24.5%, **upper secondary education**: 30.2%, **tertiary education**: 39.5%	Nether-lands	Dutch **Health Activities Literacy Scale (HALS)** (191 items): very poor skills (level 1) to very strong skills (level 4)	Participants Adult Literacy and Life Skills Survey (ALL), ≥25 years (N = 5,136)	**Health status** (general health, physical health, mental health)	HL partially mediated the relationship between education and self-reported general health, physical health and mental health (*p* < .01). HL more important among participants with lower education than among those with higher education.	G
**Mixed**								
Bennett, 2009	Cross-sectional	**Race/Ethnicity:** White: 85.3%, Black: 7.3%, Latino/Hispanic: 5.1%, Other: 2.3%; **Education:** <high school: 24.3%, = high school: 38.5%, >high school: 37.3%	USA	English & Spanish **NAAL health literacy scale: 28 health-related literacy tasks**: below basic, basic, intermediate, proficient	US adults nationally representative sample, ≥ 65 years (Racial: N = 2,668; Education: N = 2,663)	**Health status**; **Preventive health behaviors**	HL mediated relationship between racial/ethnic (black vs. white) and self-rated health status and influenza vaccination (*p* < .001). HL mediates relationship between education and self-rated health status, influenza vaccination, receipt of mammography, dental care (*p* < .001).	G
Howard, 2006	Cross-sectional	**Racial/Ethnic:** White: 87%, Black: 13%; **Education:** high school deg.: 64%, no deg.: 36%	USA	English & Spanish **S-TOFHLA:** inadequate, marginal, adequate	Elderly individuals enrolling in Medicare managed care plans in 4 different locations, ≥65 years (Racial: N = 2,850; Education: N = 3,260)	**Physical and mental health**; **General health status**; **Receipt of vaccinations**	HL reduced educational disparities for physical health (decrease of adjusted difference 0.7; 95% CI 0.4 to 0.9), mental health (decrease of adjusted difference 0.3; 95% CI 0.1–0.5), to a lesser extent for health status and very small extent for vaccination receipt. HL reduced racial disparities for physical health (decrease of adjusted difference 0.6; 95% CI 0.3 to 0.9), mental health (decrease of adjusted difference 0.3; 95% CI 0.1–0.5), to a lesser extent for self-rated health status and very small extent for vaccination receipt.	F
**Cancer-Related Outcomes**								
**First Author**	**Design**	**Participants**	**Country**	**Instrument; Cut-off points**	**Sample**	**Outcomes assessed**	**Associations**	**Quality**
**Race/Ethnicity**								
Bennett, 1998	Cross-sectional	**White**: 49%, **Black**: 51%	USA	English **REALM**: 6th vs. 3rd grade	Men at prostate cancer clinics (1.) a university hospital and (2.) VA medical center (N = 212)	**Stage of presentation with prostate cancer**	After adjustment for HL, and other covariates, race was not a sig. predictor anymore. However, also HL (OR 1.6; 95% CI 0.8–3.4) was also no longer sig. associated with stage of presentation with prostate cancer	G
Freedman, 2015	Cross-sectional	**White**: 44.5%, **AA**: 28.5%, **Hispanic**: 27%	USA	English & Spanish **Three Health Literacy Screeners** ^1^: continuous	Female breast cancer patients: population-based cohort (N = 500)	**Knowledge about one`s breast tumor characteristics**	HL reduced differences in Hispanic women (vs. white women) for knowing and correctness about their breast cancer characteristics (*p* < .05). HL did not reduce differences in black women (vs. white women). Overall HL did not eliminate most of the racial differences found.	F
Matsuya-ma, 2011	Cross-sectional	**Non-Hispanic White**: 55.1%, **AA**: 44.9%	USA	English **REALM**, **S-TOFHLA**: continuous	Newly diagnosed adults with solid tumor cancers, stages II–IV who would be receiving treatment at a cancer center (N = 138)	**Self-reported cancer information needs**	AA race was associated with greater information need but HL was not sig. associated with information needs. Educational attainment reduced the effect of race for most variables, including HL, on information needs to n.sig..	F
Wolf, 2006	Cross-sectional	**White**: 31.5%, **AA**: 68.5%	USA	English **REALM**: low marginal, functional	Men with newly diagnosed prostate cancer in outpatient oncology or urology clinics in four clinics (N = 308)	**Prostate-specific antigen (PSA) levels: medical charts**	After adjustment for HL skills and age, being black was n.sig. associated anymore with PSA levels. The inclusion of HL contributed to a reduction of 35% in the association between race and PSA level (without HL, AOR 4.6, 95% CI 2.0–9.5 vs. with HL, AOR 3.0, 95% CI 0.8–9.1)	G
Song, 2014	Cross-sectional	**Caucasian-American**: 49.8%, **AA**: 50.2%	USA	English **REALM**: <high school vs. ≥high school	Participants of a prostate-cancer population based cohort study, 1 to 27 months after diagnosis (N = 1854)	**Patient-provider communication** (content of dialogue, affective component. nonverbal behaviors)	N.sig. racial differences with regard to patient-provider communication. Sig. differences in HL between White and AA. HL (r = -0.089, *p* = .178) was not a sig. predictor in the final model, neither was race, but amongst others education (r = 0.19, *p* = .01).	G
**Mixed**								
Hovick, 2014	Cross-sectional	**Racial/Ethnic**: White: 50%, AA: 25%, Hispanic: 25%; **Education** (continuous): high school diploma: 97%; **Income** (continuous): below US-$ 20000: 24%	USA	English **NVS**: continuous	National online research panel using purposive sampling strategy (N = 1007)	**Cancer risk knowledge; Cancer risk information seeking**	HL did not mediate the relationship of SES/race and cancer risk information seeking but it mediated the effects of income, education and race/ethnicity (Hispanic, Black vs. White) on cancer risk knowledge (*p* < .01)	F
**Medication Adherence/Management** ^2^								
**First Author**	**Design**	**Participants**	**Country**	**Instrument; Cut-off points**	**Sample**	**Outcomes assessed**	**Associations**	**Quality**
**Race/Ethnicity**								
Bailey, 2009	Cross-sectional	**White**: 42%, **AA**: 58%	USA	English **REALM**: low, marginal, adequate	Adults in three outpatient family medicine clinics in (N = 355)	**Understanding of dosage instructions for a liquid medication** commonly prescribed for children	Inclusion of HL reduced the effect of race on misunderstanding to n.sig.. Marginal (AOR 2.20, 95% CI 1.19–3.97) and inadequate HL (AOR 2.90, 95% CI 1.41–6.00) remained sig. predictors of misunderstanding.	F
Osborn, 2007	Cross-sectional	**White**: 54.9%, **Black**: 45.1%	USA	English **REALM**: low (≤6th grade), marginal (7th-8th grade), adequate (9th grade),	HIV patients on one or more antiretroviral medications at two outpatient infectious disease clinics (N = 204)	**Self-reported HIV-medication adherence**	When HL was included in a regression model the effect of black race on medication adherences was reduced by 25% to non-significant (AOR 1.80, 95% CI 0.51–5.85), low HL remained a significant predictor (AOR 2.12; 95% CI 1.93–2.32)	G
Osborn, 2011	Cross-sectional	**White**: 65%, **Black**: 35%	USA	English **REALM**, **WRAT-3**: less than 9th grade, 9th grade or higher; Diabetes Numeracy Test: Quartiles	Adults with type 2 diabetes from two primary care and two diabetes specialty clinics (N = 383)	**Self-reported diabetes medication adherence**	HL was associated with adherence (r = .12, *p* < .02), but diabetes-related numeracy and general numeracy were not associated with adherence. HL diminished the effect of race on adherence to n.sig.	G
Waldrop-Valverde, 2010	Cross-sectional	**Non-AA**: 16%, **AA**: 84%	USA	English & Spanish **TOFHLA**: continuous. English & Spanish applied problems subtests of the Woodcock Johnson—III Tests of Achievement: continuous	HIV patients at HIV care clinics who were enrolled in an AIDS Drug Assistance Program (N = 207)	**Medication management capacity** (mock)	No significant differences with regard to HL found for different racial groups but for numeracy. Numeracy mediated the effect of race on poor medication management. Numeracy was significantly associated with medication management (r = 0.67, *p* < .001)	G
**Gender**								
Waldrop-Valverde, 2009	Cross-sectional	**Men**: 58%, **Women**: 42%	USA	English & Spanish **TOFHLA**: continuous. English & Spanish applied problems subtests of the Woodcock Johnson—III Tests of Achievement: continuous	HIV patients at HIV care clinics who were enrolled in an AIDS Drug Assistance Program and currently received/about to start antiretroviral treatment. (N = 155)	**Medication management capacity** (mock)	No significant differences with regard to HL found for gender but for numeracy. Numeracy mediated the relationship between gender and medication management (a: β = -0.428, *p <* .01, b: β = 0.644, *p* < .05)	G
**Disease Control**								
**First Author**	**Design**	**Participants**	**Country**	**Instrument; Cut-off points**	**Sample**	**Outcomes assessed**	**Associations**	**Quality**
**Race/Ethnicity**								
Curtis, 2012	Cohort Study	**White/Other**: 15%, **AA**: 56%, **Latino**: 29%	USA	English **REALM**: limited vs. adequate	Asthma patients, 18–40 years old: four school sampling groups (N = 348)	Six follow-up interviews on **asthma quality of life** (AQOL), **asthma-related emergency department visits, hospitalization, asthma control**	HL reduced effect of race between Latinos and Whites for quality of life and asthma control (*p* < .01) to n.sig.. HL reduced the effect of race on disparities between AAs and Whites for asthma control, ER visits and asthma quality of life to n.sig.. Only the risk for asthma-related hospitalization for AAs remained (RR = 2.97; 95 CI = 1.09, 8.12, p = .03).	G
Osborn, 2009	Cross-sectional	**White**: 65%, **Black**: 35%	USA	English **REALM, WRAT-3**: less than 9th grade, 9th grade or higher; **Diabetes Numeracy Test**: Quartiles	Adults with type 2 diabetes from two primary care and two diabetes specialty clinics (N = 398)	**Glycemic control** (Chart review: most recent A1C value)	HL and general numeracy n.sig. predictors of glycemic control. Diabetes-related numeracy (r = -0.17, *p* < .01) diminished the effect of AA race on glycemic control to n.sig..	G
Sperber, 2013	RCT	**White**: 54%, **Black**: 43%	USA	**REALM**: low vs. high	Participants enrolled in primary care at a VA medical center with diagnosis of hip and/or knee osteoarthritis and persistent, current self-reported joint symptoms (N = 461)	Effects of a 12-months telephone-based osteoarthritis self-management support intervention: **Arthritis outcomes**	In the telephone-based osteoarthritis (OA) self-management support intervention compared to the usual care arm (*p* < .05) a sig. interaction effect for race and HL on change in pain was found; non-whites with low HL in the intervention had the highest improvement in pain. For mobility, walking and bending, affect, general pain and self-efficacy no sig. effects were found.	G
**Education**								
Pandit, 2009	Cross-sectional	**grade 1–8**: 15.5%, **grade 9–11**: 15.5%, **= high school**: 36%, **> high school**: 33%	USA	English **S-TOFHLA**: five categories	Patients with diagnosed hypertension and scheduled appointments at six primary care safety net clinics (N = 289)	**Hypertension knowledge and control**	When HL was added to models that included only education, the association between education and knowledge was diminished to n.sig. (Grades 1–8: β = -.30, 95% CI -1.44–0.83), whereas the association between education and hypertension control was only minimally reduced (AOR 2.46, 95% CI 2.10–2.88). Limited HL was associated with hypertension control in the final adjusted model (AOR 2.68, 95% CI 1.54–4.70). No sig. interaction effects were found.	G
Schillinger, 2006	Cross-sectional	**<high school graduate**: 46.8%, **high school graduate or GED**: 24.1%, **technical school or college attendance or graduation**: 29.1%	USA	English & Spanish **S-TOFHLA**: continuous	Type 2 diabetes patients from two primary care clinics (N = 395)	**Glycemic control** (Chart review: most recent A1C value)	HL sig. mediated the effect of education on A1C (*p* < .05), the direct association between education and A1c diminished to n.sig..	G
**Preventive Care** ^3^								
**First Author**	**Design**	**Participants**	**Country**	**Instrument; Cut-off points**	**Sample**	**Outcomes assessed**	**Associations**	**Quality**
Lindau, 2002	Cross-sectional	**White**: 14%, **AA**: 58%; **Hispanic**: 18%, **Other**: 10%	USA	English **REALM**: inadequate, marginal, adequate	Women in ambulatory women`s clinics at an academic medical center (N = 529)	**Cervical cancer screening history and knowledge**	When adjusting for HL, ethnicity was not a sig. predictor of cervical cancer screening knowledge (AOR 2.25; 95% CI, 1.05–4.80). No racial differences with regard to behavioral variables found.	F
Sentell, 2013	Cross-sectional	**White**: 91% (1% LEP), **Asian**: 9% (33.5% LEP)	USA	English, Spanish, Chinese (Mandarin, Cantonese), Korean, and Vietnamese: **two subjective proxy measures of health literacy**: continuous.	Participants 2007 California Health Interview Survey (CHIS) 50–75 years (N = 15,888)	**Compliance with colorectal screening guidelines**	Low HL only was not a sig. predictor among Asians (OR 0.71, 95% CI 0.39–1.28) for meeting colorectal cancer screening guidelines but LEP-only was a sig. predictor (OR 0.62, 95% CI 0.38–0.99). Both LEP and low HL was sig. associated with having a lower likelihood of cancer screening (OR 0.50, 95% CI 0.28–0.89).	F
**End-of-Life Decisions**								
**First Author**	**Design**	**Participants**	**Country**	**Instrument; Cut-off points**	**Sample**	**Outcomes assessed**	**Associations**	**Quality**
Sudore, 2010	Cross-sectional	**White**: 25%, **AA**: 24%, **Latino**: 31%, **Asian/Pacific Islander**: 9%, **Multiracial**: 10%	USA	English & Spanish **S-TOFHLA**: limited vs. adequate	General medicine outpatients in a county hospital ≥50 years (N = 205)	**Decisional uncertainty about making advance treatment decisions**	Adjusted analysis: adequate HL (AOR 2.11, 95% CI 1.03–4.33), being Latino (AOR 2.50, 95% CI 1.01–6.16) or Asian-Pacific Islander (AOR 4.25, 95% CI 1.22–14.76) vs. White remained independently associated with uncertainty about treatment (Black was not associated at all). Magnitude of effect of race did not change significantly when HL was added to the model.	F
Volandes, 2008	Cross-sectional experimental study	**White**: 44%, **AA**: 56%	USA	English **REALM**: low, marginal, adequate	Patients scheduled to see a general internist: at six primary care clinics; ≥40 years (N = 144)	**End-of-life care preferences**	Before experimental stimulus: Adjusted analysis: HL mediated the relationship between race and end-of-life preferences for African-Americans (Low HL: AOR 7.3, 95% CI 2.1–24.2; Marginal HL: AOR 5.1, 95% CI 1.6–16.3)	F
Waite, 2013	Cross-sectional	**White**: 56.9% (6.9% “other”), **AA**: 43.4%	USA	English **TOFHLA**: inadequate, marginal, and adequate	Participants at one academic general internal medicine clinic or four health centers; 55–74 years (N = 784)	**Having an advance directive**	Introduction of HL (low HL: RR 0.45, 95% CI 0.22–0.95) into multivariable model reduced influence of race but AA race remained sig. associated (RR 0.64, 95% CI 0.47–0.88) with having an advance directive.	G
**Other Health Outcomes**								
**First Author**	**Design**	**Participants**	**Country**	**Instrument; Cut-off points**	**Sample**	**Outcomes assessed**	**Associations**	**Quality**
**Race/Ethnicity**								
Bains, 2011	Cross-sectional	**White**: 56%, **AA**: 41%, **Hispanic/Other**: 3%	USA	English **REALM-R**: inadequate vs. adequate	Patients at an adult primary care clinic (N = 347)	**Usage of complementary and alternative medicine (CAM)**	Sig. interaction between race and HL. Whites with adequate HL were more likely to use CAM (adjusted OR 9.42, 95% CI: 1.66–53.5, *p* = .01) but in AAs adequate HL was not sig. related to CAM usage (adjusted OR 0.97, 95% CI: 0.27–3.48).	F
Langford, 2012	Cross-sectional	**White**: 81%, **Black**: 10%, **Hispanic**: 9%	USA	English & Spanish **Numeracy: three subjective measurements (HINTS 2007):** Likert-type scale	Health Information National Trends Survey (HINTS): nationally representative sample (N = 6,754)	**Awareness of Direct-to-consumer (DTC) genetic tests**	When two numeracy variables were added to the model, the effect of black (vs. white) was no longer sig. (OR = 0.84; CI 0.69–1.04). Hispanics did not sig. differ from Whites with regard to DTC genetic tests awareness. No sign. interaction of race/ethnicity with SES and numeracy variables DTC genetic tests awareness.	F
Smith, 2012	Cross-sectional	**English-speaking**: 50%, **Spanish-speaking**: 50%	USA	English & Spanish **TOFHLA**: low, medium, high	Patients in an ED who had received instructions for a follow-up appointment and/or medication refill within one week (N = 100)	**Adherence to ED discharge instructions**	Spanish-speaking participants with low level of HL were sig. less likely than English-speakers to show up for follow-up appointments (*p* < .001). Spanish-speaking participants with high HL level were more likely than the other groups to have understood their discharge instructions.	P
Gardiner, 2013	Cross-sectional	**Non-Hispanic White**: 29%, **Non-Hispanic Black**: 52%, **Hispanic/other race**: 19%	USA	English **REALM**: low vs. high	Patients in an inner-city hospital (N = 581)	**Usage of complementary and alternative medicine (CAM)**	Sig. interaction found between HL and race for any CAM use and for provider-delivered therapies. Use of any CAM among White (OR 3.68, 95% CI 1.27–9.9) or Hispanic/other race (OR 3.40, 95% CI 1.46–7.91) was sig. higher among those with higher HL. Hispanics/other race with higher HL were more likely to use provider-delivered therapies (OR 3.59, 95% CI 1.27–10.19).	F
**Mixed**								
Mottus, 2014	Cross-sectional	**Education:** No qualification: 17%; O-level: 39%, A-level: 16.3%, (Semi)professional: 12.2%, University degree: 15.5%; **Occupational social class:** Unskilled: 0.5%, Semiskilled: 3.4%, Skilled manual: 16.6%, Skilled non-manual: 21.5%, Intermediate: 38.2%, Professional: 19.7%	Scotland	British versions of: **REALM, S-TOFHLA passage B, NVS**: all continuous combined into a latent HL factor	Lothian Birth Cohort 1936 –participants at around age 73 years (N = 730)	Three objective health outcomes in older people: **General physical fitness**; **BMI**; **Number of natural teeth**	Lower HL was linked to worse health outcomes, but educational and occupational level, as well as cognitive abilities, accounted for most of these relationships. After adjusting for covariates (including education and occupation), only physical fitness was significantly associated with HL.	G
Yin, 2009	Cross-sectional	**Child disparities—Parent’s education:** Still in school: 0.5%, <High school: 13.7%, = High school: 29.5%, >High school: 56.3%; **Race/Ethnicity:** Non-Hispanic White: 66.1%, Non-Hispanic Black: 12.2%, Hispanic: 16.1%, Other: 5.7%; **Income:** < Poverty threshold: 18.2%, 100%-175% of poverty threshold 16.25%, >175% of poverty threshold: 58%; **English Proficiency:** Understands very well: 83.1%, Understands well: 10.8%, Understands not well/not at all: 6.1%	USA	English & Spanish **NAAL health literacy scale**: 13 out of 28 health-related literacy tasks: Below basic, Basic, Intermediate, Proficient	Parents of children—nationally representative sample (N = 6,100)	**Child health insurance status**; **Difficulty understanding OTC-medication labels**; **Food label use**	After inclusion of HL (below basic HL: OR: 2.4, 95% CI 1.1–4.9) education and race/ethnicity was no longer a sig. predictor of health insurance status. Education, race/ethnicity and income were no longer significant after including HL (below basic HL: OR: 3.4, 95% CI 1.6–7.4) in predicting understanding of OTC medication labels. HL was n.sig. related to food-label use.	F

Significant (sig.); Non-significant (n.sig.); Limited English Proficiency (LEP); African-American (AA); Categorizations of race/ethnicity reported as in the studies.

^1^ Chew et al., [28]

^2^Yin et al. [48] reported on “Medication Adherence & Management” *and* “Other Outcomes”.

^3^Bennett, Chen, Soroui, & White, S. (2009); Howard, Sentell, & Gazmararian (2006) reported on “Self-reported Health Status” *and* “Preventive Care”

### Self-reported health status

Eight studies investigated in how far health literacy might explain racial disparities in self-reported health status, including mental health.

Two studies found that health literacy mediated or respectively reduced the effect of race/ethnicity (black vs. Hispanic vs. white) and education on self-reported general health [30], including physical and mental health [31]. Three studies explored the link by focusing on Asian American groups and found mixed evidence. In one study, health literacy was significantly associated with self-reported health status and depression symptoms in White and Asian immigrants in general. However, when disaggregated in separate groups, health literacy was only significantly associated with self-reported health status in Chinese and Korean participants [32]. Similarly another study found that low health literacy was significantly associated with poor health status in Japanese and Filipinos, as well as in White participants [33]. On the other hand, another study found that limited English proficiency (LEP) was a more important predictor than health literacy in explaining differences in self-reported health status in Hispanics, Vietnamese, Whites and “other races”. Low health literacy was only significantly related to health status in Whites and “other races” but not in any Asian group. Further, Chinese, Vietnamese, Hispanics and “other races” with low health literacy *and* LEP had the highest odds of poor health status [34]. Similar patterns were identified in Canada (immigrants vs. non-immigrants) [35]. Even though health literacy was significantly associated with good self-rated health, discordance between native language and language of data collection reduced the effect of health literacy to non-significance. Similarly, another study from Canada found that among different generations of immigrants, health literacy was significantly associated with reported disability but the effect was largely accounted for by differences in education, employment and income [36].

In one study from China, which compared two ethnic groups (Han vs. Hui), health literacy was significantly associated with prevalence of pain in a minority group. There was also a significant interaction effect between health literacy and ethnicity on self-reported anxiety/depression [37].

Only one study from the Netherlands looked specifically into educational disparities, and discovered that health literacy partially mediated the relationship between education and self-reported general health [38].

### Cancer-related outcomes

Six studies investigated how health literacy might explain disparities in cancer-related outcomes. Three studies looked into the effect of race/ethnicity (black vs. white) and health literacy in prostate cancer patients [39–41]. One study found that, after adjustment, race/ethnicity and health literacy were no longer significant predictors of presentation with advanced stage prostate cancer [39], whereas another study found that health literacy contributed to a reduction of 35% in the association between race/ethnicity and prostate-specific antigen levels [41]. With regard to disparities in patient-provider communication in a sample of patients with prostate cancer, Song and colleagues [40] did not find any significant relationship between race/ethnicity and health literacy and in the final model health literacy was not a significant predictor.

An additional two studies found that education was a more important predictor for information needs in newly diagnosed cancer patients [42] and cancer risk information seeking [43] than health literacy. Yet, health literacy was a significant mediator between SES and race/ethnicity on cancer risk knowledge [43].

One study investigated how health literacy might influence racial/ethnic differences (black vs. Hispanic vs. white) in female patients’ knowledge about their breast cancer characteristics, and found that health literacy did not eliminate most of the racial/ethnic differences under investigation. However, health literacy reduced differences between White and Hispanic women for being accurately knowledgeable about their breast cancer characteristics [44].

### Medication adherence & management

Health literacy mediated the effect of race/ethnicity (black vs. white) on HIV and diabetes medication adherence in two studies. However, the studies did not find a significant relationship between diabetes-specific and general numeracy on medication adherence [45, 46]. In another study, the inclusion of health literacy as a predictor reduced the effect of race/ethnicity (black vs. white) on understanding of dosage instructions for pediatric liquid medication [47]. These results concur with Yin and colleagues [48] results, where after including health literacy in the final model, neither race/ethnicity, nor education or income predicted the understanding of OTC medication labels.

Yet two other studies discovered that numeracy mediated the effect of race/ethnicity (black vs. white) and gender on medication management capacities in HIV patients [49, 50].

### Disease control

Five studies looked into disease control (e.g. asthma-related hospitalizations or glycemic control in diabetes patients). One study found that diabetes related numeracy diminished the effect of race/ethnicity (black vs. white) on glycemic control to a level of non-significance. However general health literacy or numeracy were no significant predictors of glycemic control [51]. Another study demonstrated that health literacy reduced the effect of race/ethnicity in African-Americans and Hispanics on asthma quality of life and asthma control, and for African-Americans only on emergency department visits. However, differences between Afro-Americans and Whites for asthma-related hospitalizations remained [52].

Overall only one experimental study looked at the differential effects of race/ethnicity (black vs. white) and health literacy. The telephone-based osteoarthritis (OA) self-management support intervention found a significant interaction effect between health literacy and race/ethnicity on change in pain. Non-Whites with low health literacy had the highest improvement in pain in the intervention group compared to the usual care group [53].

Two studies looked into educational disparities. Whereas in one study health literacy significantly mediated the effect of education on glycemic control [8], in the other study health literacy reduced only minimally the effect of education on hypertension control but reduced it to non-significance for hypertension knowledge [54].

### Preventive care

Three studies investigated the influence of health literacy on cancer screening behavior and knowledge. One study did not find significant racial/ethnic (black vs. Hispanic vs. white vs. other) differences with regard to behavioral variables. Yet, after adjusting for health literacy, race/ethnicity was no longer a significant predictor of cervical cancer screening knowledge [55]. Another study in Asian Americans found that low health literacy was not significantly associated with meeting colorectal cancer screening guidelines in the Asian group. LEP was a more important predictor, as well as the combination of LEP *and* low heath literacy [56].

Bennett and colleagues [30] discovered that health literacy mediated the relationship between education and receipt of a mammography. Also, they found that it mediated the effect of educational disparities on dental care and educational and racial disparities on receipt of influenza vaccine.

One study looked into receipt of vaccinations, in which health literacy reduced the influence of race/ethnicity and education on vaccination receipts only minimally [31].

### End-of-life decisions

Three studies investigated the relationship between health literacy and end of life related decisions. One study found that, before an experimental stimulus was introduced to the participants, health literacy reduced the association between race and end-of-life preference to non-significance [57]. On the other hand, in two other studies, even though health literacy was significantly associated with decisional uncertainty about making an advance treatment decision [58] or having an advance directive [59], it did not significantly reduce the effect of race/ethnicity.

### Other health outcomes

Two studies looked into racial/ethnic disparities (black vs. Hispanic/other vs. white) in the usage of complementary and alternative medicine (CAM), and both found a significant interaction effect between race/ethnicity and health literacy. In both studies, Whites with higher levels of health literacy were more likely to use any kind of CAM [60, 61]. Gardiner and colleagues [61] identified the same effect in Hispanics, as well as a higher likelihood to use provider-delivered therapies. This was not the case for the relationship in black participants.

One study looked into how numeracy was related to direct-to-consumer genetic testing, in which the addition of numeracy to the final model reduced the effect of black race to non-significance. On the other hand, Hispanics did not significantly differ from White participants. There were no significant interactions between race/ethnicity and numeracy [62].

Overall, this review identified only one study that evaluated the relationship between health literacy and physiological outcomes as an indicator of general health status. The study, conducted in an elderly population in Scotland, found that health literacy was linked to worse health outcomes. However, educational and occupational level, as well as cognitive abilities accounted for most of the relationships. Only physical fitness was significantly related to health literacy after adjusting for educational and occupational level and other variables [63].

Yin and colleagues [48] investigated in how far parents’ health literacy mediated the effect of a variety of potential disparities on outcomes relevant to their children. However, after the inclusion of health literacy in the final model, the association of race/ethnicity and health insurance status was no longer significant. As already described above, only understanding of OTC medication labels was significantly associated with parents’ below basic health literacy.

Another study compared Spanish- to English-speakers in an emergency department and concluded that the former were less likely to keep up follow-up appointments if they had lower health literacy [64].

## Discussion

In the conceptual literature health literacy is commonly described as a contributing factor to health disparities but little is known about its actual contribution and potential role in the relationship between social and health disparities. This review identified overall 36 studies that have investigated this relationship. Most of these studies focused on racial/ethnic disparities, followed by some few studies that systematically looked into educational disparities. Only one study specifically investigated the contribution of health literacy on potential gender differences in health.

Given that nearly all studies were conducted in the US, the large focus on racial/ethnic disparities in the identified studies is not surprising. Race/ethnicity is often an assumed proxy for other variables. Whether race is a biological category as such, an indication of socioeconomic status or an independent predictor in which socioeconomic status solely is a mediator, studies on health disparities in the US have largely used race/ethnicity as a predictor of health outcomes [65].

Overall the review showed that the evidence on the role of health literacy on disparities is still mixed and, for most outcomes, very limited. Even though race/ethnicity was a commonly investigated social disparity, outcomes under investigation and measurements largely varied in the identified studies. Thus, making direct comparisons between different studies rather difficult. There were no evident patterns on whether some health literacy measures were more predictive than others in the relationships under investigation. As suggested for clinical settings, measures of functional health literacy, such as the REALM or the S-TOFHLA, had been most frequently used when evaluating the relationship in patients. On the other hand, when investigating the relationship in population-based data, relatively often subjective health literacy measures were used.

Overall, some very limited evidence was found on the mediating function of health literacy on self-rated health status when looking into educational disparities. Further, some evidence was identified on the role of health literacy as an independent predictor of health status across different racial/ethnic groups, which showed to be a constant relationship independently from health literacy measures used. However, results need to be carefully evaluated given that the racial/ethnic groups under investigation largely varied from one study to another. There was also some limited evidence of the potential effect of health literacy, in particular numeracy, on reducing racial/ethnic disparities on medication adherence and understanding of its intake. Further, some evidence was found on the effect of health literacy on knowledge-related outcomes.

With regard to cancer-related outcomes, the included studies yet failed to show any patterns. Four out of six studies did not find health literacy to be a significant predictor in the relationships under investigation.

Part of the explanation for the differences found for self-reported heath status on the one side and cancer-related outcomes on the other side, might lie in the fact that some of the cancer-related outcomes were not related to actual health outcomes but related concepts, such as information seeking/needs, presentation stage or doctor-patient communication. This in turn might be related to other, in this case, more important mediating mechanisms, such as self-efficacy for instance [66–68]. The role of other mediating mechanisms indeed has to be carefully evaluated. This review found, for example, that in the case of immigrants language proficiency was more important in predicting differences in health than health literacy. Other studies, even though not with the focus on examining health disparities, have found similar patterns when scrutinizing the relationship between health literacy and health(-related) outcomes and found that other variables, such as the above-mentioned self-efficacy or knowledge were indeed important mediating variables [69–71].

Even though two independent reviewers rated the quality of most studies as “fair”, there are still some limitations that are inherent to a number of the included studies. Overall, most of the data sets that had been used in the studies originally had not been collected to investigate the relationship between health literacy and disparities per se, but also to investigate a variety of other relationships. Hence, some of the results presented in these studies initially might have been of secondary interest and were not necessarily built on previous results and literature. This might explain some of the incongruences between the different studies. Further, from a conceptual perspective, studies often neglected to sufficiently describe the disparity under investigation. General assumptions were made on how health literacy should contribute to disparities but the disparity as such was often only vaguely described. Moreover, descriptions of the disparities under investigation often primarily focused on discussing health literacy as a social determinant of the disparity tested. Even though the conclusion that health literacy might be a determinant per se in this relationship is certainly not wrong, it does not sufficiently take the possible pathways on how health literacy influences health disparities into account. This is also mirrored in the fact that only few studies tested for predefined hypotheses, in which the possible pathways were described. A couple of studies made assumptions about its mediator role, testing for it by using appropriate analysis techniques, including mediation analysis and estimating structural equation models. Other studies tested its potential moderator role and tested, for example, for interaction effects. Still, in most of these studies the potential role of health literacy was not specifically predefined and left a lot of room for interpretation.

**Fig 3:**
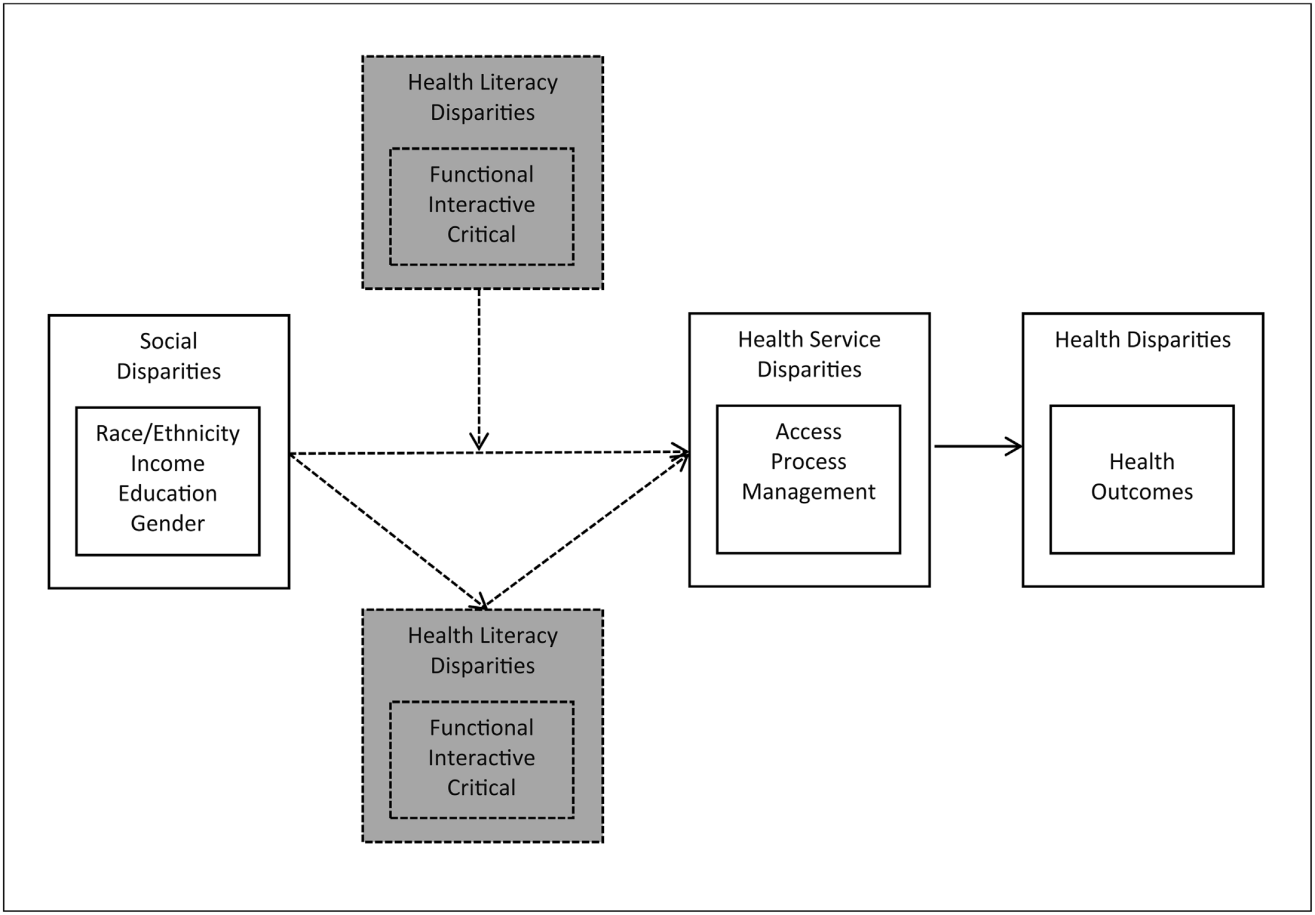
Possible pathways on how health literacy explains disparities in health outcomes.

Potential pathways might distinguish between social disparities, including race, income or education, and a potential “health literacy disparity” as such (Fig 3), thereby trying to identify not only research gaps but also potential leverage points for interventions that aim at reducing disparities. It is indeed here that the conceptualization and measurement of health literacy might need to move beyond the functional dimension, and instead focus more systematically on other dimensions such as interactive and critical literacy [72]. By taking these dimensions into account, one would be able to identify crucial points that are key to, for example, accessing and processing health services and information needed. This also has implications from an interventionist point of view. It would provide clear indications on how and where to increase efforts to reduce health disparities by means of health literacy related interventions, such as providing simplified access to information or support during health-related decision making.

### Limitations

The synthesis presented in this review needs to be carefully evaluated. Studies included in this review used different health literacy measures, cut-off points and analysis techniques, which made comparability sometimes difficult. Further, even though all studies sufficiently controlled for covariates, there are still inconsistencies between studies. Due to this heterogeneity it was not possible to perform a meta-analysis. Also, despite the attempt to be as comprehensive as possible in the search strategy, some studies might not have been included in this review. Moreover, the search was limited to already published material, thus potentially missing any work that is currently in preparation or under evaluation for publication.

Included in the review were only studies that explicitly acknowledged the disparity under investigation, therefore ignoring any study that evaluated the relationship between literacy and disparity as a secondary outcome. Future reviews might therefore need to focus on smaller characteristics, such as race/ethnicity or education, to fully grasp the relationships leading to health disparities. In addition, as the field of health literacy is still evolving, consensus on which dimensions should be included when assessing health literacy is still lacking. Therefore, studies that primarily assessed health knowledge, including mental health literacy, were excluded. Only studies that explicitly acknowledged health literacy and included a valid measure of health literacy were included.

### Conclusions

Evidence on the exact nature of the relationship between health literacy and health disparities remains still scarce. Most studies identified in this review focused on racial/ethnic disparities, being a proxy for other important predictors of health disparities. Some limited evidence was found on the role of health literacy in mediating educational and racial/ethnic disparities with regard to self-reported health status. Also, some evidence was found on its role as a mediator between racial/ethnic disparities and medication management/adherence and health knowledge. Only few studies tested for hypothesized pathways and systematically scrutinized the relationship between health literacy and health disparities. There is a need to systematically conceptualize the pathways that link health literacy to health disparities and to address whether other social disparities interfere with this relationship. Especially longitudinal studies would shed more light on the potential causal pathways explaining the potential mediating function of health literacy and other mediating variables on health disparities. More rigorous studies are needed that not only clearly define the disparity but also the groups under investigation, by choosing an appropriate reference group and potentially holding other social factors constant between these groups.

## Supporting Information

S1 Appendix PRISMA Checklist. (DOC)

S2 Appendix Full search strategy PubMed/Medline. (TIFF)
