# Supplementary figures and images for: The Relationship between Health Literacy and Health Disparities: A Systematic Review
Source: PLoS One. 2015 Dec 23;10(12):e0145455. doi: 10.1371/journal.pone.0145455 (PMC4689381; doi:10.1371/journal.pone.0145455)

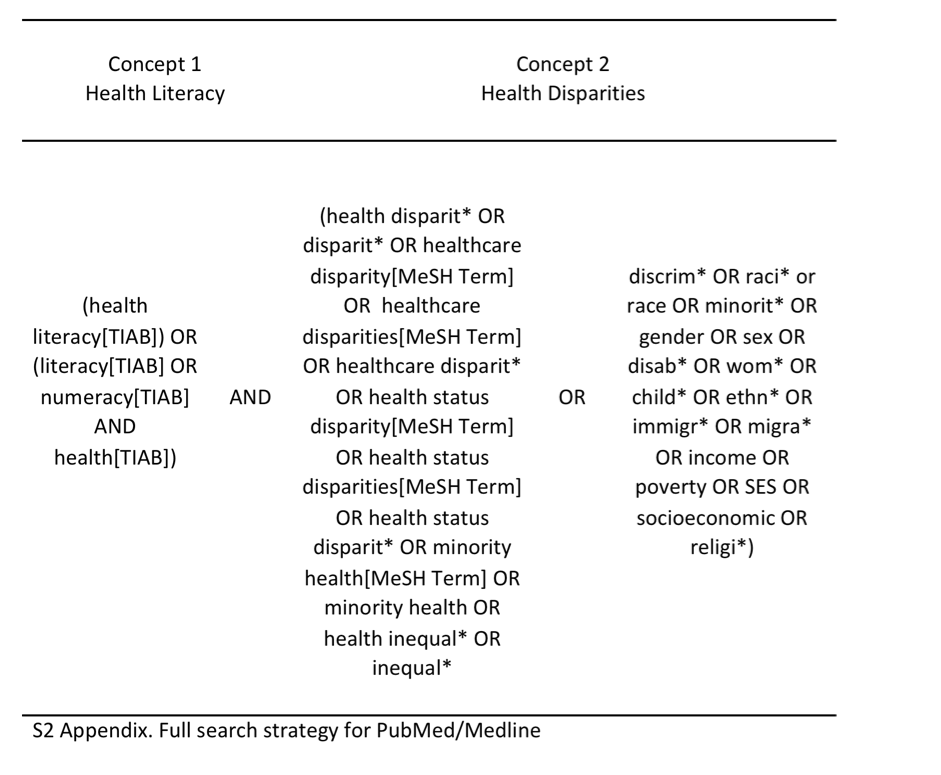

Supplement: S2 Appendix — (TIFF) [file pone.0145455.s002.tiff]
